# Tourism Management Strategies under the Intelligent Tourism IoT Service Platform

**DOI:** 10.1155/2022/7750098

**Published:** 2022-04-12

**Authors:** Guo Chen

**Affiliations:** School of Tourism and Management, Xinyang Agriculture and Forestry University, Henan 464100, China

## Abstract

This paper conducts an in-depth study and analysis of the tourism management strategy of the intelligent tourism IoT service platform. The intelligent tourism system is designed from the overall system architecture to the design of each functional module of the system, and the database design is completed with the help of the database E-R diagram. At the same time, this paper uses a weighted regression mathematical method to analyze the passenger flow at different stages of the scenic spot by establishing a scenic spot passenger flow prediction model and giving the results of passenger flow prediction for the scenic spot in the next seven days. In addition, this paper conducts a research based on the ant colony algorithm to establish an itinerary planning and recommendation model to recommend an itinerary that meets the needs of scenic tourists and provides reliable tour guidance based on their expected touring time and touring expenses in the scenic area. The method simplifies the solution of service combination problems in the IoT environment by transforming them into service instance preferences within abstract services. The effectiveness of the method is demonstrated through theoretical derivation. On this basis, the residual energy and energy consumption of smart terminal devices abstracted as cumulative aggregation QoS attributes, and an energy consumption balancing service combination method based on cumulative aggregation QoS is proposed. The countermeasures and suggestions are proposed to increase tourism publicity and give full play to the advantages of tourism resources; strengthen tourism environment monitoring and improve the legal and regulatory system; raise tourism construction funds and strengthen infrastructure construction; improve residents' participation and promote healthy tourism development, as well as to make long-term planning and innovate the management mode; establish a coordinating body and improve the supervision system; establish government macro-control and formulate regulations; and strengthen the management function and the management optimization strategy of cultivating grassroots organizations. Through experimental comparison with other methods, the results show that the method ensures the lifecycle and stability of the entire service portfolio workflow while providing low-energy portfolio services.

## 1. Introduction

Smart tourism is a new tourism industry that applies technologies such as the Internet of Things, cloud computing, next-generation communication networks, high-performance information processing, and intelligent data mining to modern tourism services and tourism industry management, so that tourism physical and information resources can be highly systematically integrated and deeply developed and serve tourists, tourism enterprises, and government management departments in a future-oriented way. This definition fully reflects the characteristics of modern smart tourism, which mentions the application for the government management sector, that is, smart tourism government affairs [1]. At present, domestic innovation in smart tourism is mainly in the use of commercial software, such as Baidu Maps, where to go, Crip, and other mobile phone clients, while relatively little innovation has been explored in smart tourism government. However, much of the information currently available to governments is first-hand, and many governments abroad have opened their data for commercial software to read and use, so that tourists can make timely adjustments to their travel plans based on this information. Recommend a play route that meets their needs for tourists in the scenic spot and provide reliable play guidance. The method transforms the service composition problem into the optimization of service instances in the abstract service, which simplifies the solution of the service composition problem in the Internet of Things environment [2]. As a result, the need for trustworthy tourism information released by official platforms has become an urgent need for tourism demand groups, especially self-guided travelers.

Facing the trend of integration and development of tourism and information technology, this paper, on the basis of defining the connotation and characteristics of wisdom tourism and wisdom tourism services, further clarifies the participating subjects, elements, processes, and demands of wisdom tourism services, analyzes the characteristics, functions, architecture, and development stages of the wisdom tourism network platform, reveals the evolution mechanism of wisdom tourism services based on the network platform, constructs the wisdom tourism services based on the network platform mode, and design its operation mechanism and management strategy, aiming to establish a set of systematic and scientific theoretical and methodological system of wisdom tourism service management with the network platform as the relying carrier and technical support, so as to improve the collaborative service capability of the whole tourism industry, further enhance the comprehensive advantages of the tourism industry, and promote the sustainable development of the tourism industry [3]. On the one hand, with the vigorous development of scenic wisdom tourism, scenic areas have made certain achievements in the construction of wisdom management, wisdom marketing, and wisdom services, but there are still some problems, such as the timeliness and accuracy of the managers of all aspects of the scenic area to obtain information about the scenic area that is not enough, and the scenic area involves numerous departments and resources; how to effectively integrate resources and give full play to the scenic area information technology in the management of the scenic area construction of the comprehensive effect still needs to be addressed through effective methods [4]. The problems that exist for intelligent scenic tourism can be solved by making full use of new network technology tools such as mobile Internet, Internet of things, and cloud computing, to solve the pain points and needs of scenic tourists and make the whole journey of scenic tourists more intelligent, humanized, specialized, and refined. To ensure interoperability between architectures and to provide users with greater granularity of combined services, many scholars have directly applied Web service specifications and traditional service combination methods to the IoT, but this approach is not particularly rational. In the IoT, services are often heterogeneous, resource-constrained, dynamically changing, and massive in scale. These characteristics make services in the IoT environment different from traditional Web services, so it is important to explore the service combination methods in the IoT environment.

## 2. Related Works

Based on the resource-constrained characteristics of IoT services, information such as the residual energy and geographical location of a service is used as the service QoS, and a related QoS model is constructed [5]. Service energy is introduced into the workflow management of wireless sensor networks, a scheme for calculating service energy is proposed, and the traditional service QoS is extended to achieve efficient local service selection by decomposing global constraints into local constraints for atomic services using a QoS constraint decomposition approach and a comprehensive consideration of service energy and QoS. The optimal balance problem between QoS energy consumption of IoT services is transformed into a multiobjective problem, and a pulse algorithm is proposed for its solution. The software enables visitors to experience through sound and electricity [6]. When visitors are in a place, they use a mobile camera to target ancient ruins or ruins, GPS, and maps on a mobile phone [7]. The image recognition software locates the site, restores the original appearance of the historic site, and reconstructs incomplete parts of the site. In addition, the software has a route planning function and customizes travel plans through route planning. The information processing technology put into use in a European city builds big traffic data into a wireless communication network and focuses on processing data from tourism information systems and vehicle control systems.

Smart tourism is an advanced stage in the integration development and synergistic evolution of information technology and the tourism industry [8]. Through the research of many scholars from different perspectives over the years, firstly, smart tourism is an important innovation in the tourism industry; secondly, the innovation path is based on the efficient flow and effective integration of tourism information in the tourism industry; again, the innovation means is the application of new-generation information technology such as the Internet, big data, and cloud computing. Finally, the purpose of innovation is to further enhance tourism service levels and tourist satisfaction [9]. Therefore, this paper compares the relevant literature on the concept, characteristics, structure, driving factors, development models, service ecosystems, and their evaluation of smart tourism. The tourism industry has begun to shift from the traditional tourism industry development model to the development model of smart tourism, which widely applies modern new information technology to achieve real-time sharing of tourism information and further meet the requirements of the development of smart scenic spots [10]. The four major manifestations of smart tourism are “smart business, smart management, smart services, and smart government,” to provide ideas for the construction and development of smart tourism. Government departments in charge of tourism at all levels are the primary promoters of the construction of smart tourism and are also the main responsible bodies for the construction and development of smart tourism. Tourism wisdom government refers to the tourism authorities at all levels of government through the application of wisdom technology in all aspects, to improve the government's management level and service capacity of the tourism industry, to promote better and faster development of the tourism industry [11].

The application of cloud computing in tourism wisdom government is mainly to promote the construction and application of tourism wisdom government cloud platform as a breakthrough, through the platform to achieve the wisdom of tourism government business, the maximum for tourists and most tourism service enterprises to provide technical protection and service support. Intelligent perception technology provides help for smart tourism marketing, pointing out that the recommendation system mounted on the cloud computing platform can efficiently process data and quickly calculate recommendation information, also introducing the principle of information push implementation and the technology of pushing information to mobile terminals by common servers in the platform; the multiplatform solution of cloud computing optimizes tourism informatization construction, tourism informatization construction is based on the cloud computing platform and, in practice, must gradually design and implementation of its tourism perception system, cloud data center, cloud computing platform, cloud computing application support platform, and several platform applications.

## 3. Analysis of Tourism Management Strategies for Intelligent Tourism IoT Service Platforms

### 3.1. Intelligent Tourism IoT Service Platform Design

**Figure 1:**
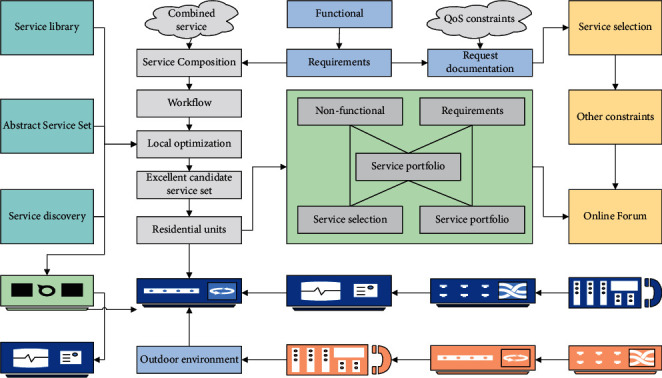
IoT service combination process analysis framework.

In the high-density IoT environment, services often present characteristics such as large scale, limited resources, single function, and service heterogeneity. However, with the increasing demand of users, a single IoT service is not good enough to meet user needs, so the service combination technology is particularly important [12]. Service combination is the process of aggregating multiple services with simple structure and single function into a complex structure and powerful combination service through a certain combination mode. The combination process of IoT services mainly includes five stages: user requirement analysis, service discovery, local optimization, service selection, and service combination, and the overall process analysis framework is shown in Figure 1.

The system design phase requires the completion of the transformation from the requirements analysis phase to a system-level solution, a process that can be achieved by applying class and package diagrams in UML. The transformation provides access to all the design classes and packages required for the system. At the same time, the design of the class interfaces can be completed. People can learn more information through mobile phones and other platforms, and the fragmentation and complexity of information make people spend time and cost in information screening, and especially in recent years, false and wrong information has been continuously forwarded by people and disturbed people's correct judgment of things. This is the core of the full lifecycle. The intelligent analysis and decision management platform allows for the measurement of the number of visitors entering the scenic area and real-time traffic flow at any time, facilitating the control of the dynamic flow of the scenic area, the assessment of potential risks, and emergency response to various security situations, as well as prediction and prevention, the statistical analysis of the tourism structure, the planning of dynamic fares for tourist attractions, and the decision to support the in-depth development of tourism resources.

(1) $$\begin{array}{c} S=\left\{\left(x_{1}^{s},y_{1}^{s}\right),\ldots ,\left(x_{1n}^{s},y_{1n}^{s}\right)\right\},\\ T=\left\{\left(x_{1}^{t},y_{1}^{t}\right),\ldots ,\left(x_{n}^{t},y_{n}^{t}\right)\right\}. \end{array}$$

Through an intelligent service platform, the processed information can be actively pushed to the tourists or the competent authorities. Tourists can automatically access the information they want to know about the relevant tourism products, such as name, gender, and age, on the public portal for scenic tourism, and the authorities can access information on the flow of people and traffic at any time. Visitors can browse the Scenic Tourism public portal, which pushes out local seasonal itineraries and tourism products based on their location and time online. For registered users, interest recommendations and interface information can be automatically optimized according to individual characteristics. Self-service tourism information such as real-time information on tourist routes, traffic flow of people nearby, parking locations near destinations, and tourist route guides can be provided [13]. Differentiated services can also be implemented for registered members. In addition, visitors can also download self-service client software from various electronic devices such as mobile phones, tablet PCs, and other terminals to obtain information on attraction spending, dining and entertainment, and travel route optimization. Other value-added consumption items can also be realized according to visitors' needs.

(2) $$\begin{array}{c} g\left(x\right)=w^{T}\theta x^{2}. \end{array}$$

The system uses as many of the most advanced domestic and international mainstream network technologies and equipment as possible, in line with the current direction of management development, to ensure that the technologies and equipment are available with practical application cases. The use of mature equipment and technology also reduces the risk of the system and minimizes losses. To ensure return on investment, reduce costs, reduce investment in research and development, and obtain higher returns, we have to choose the appropriate equipment and technology under the premise of conforming to the specifications, adopt a network topology suitable for the characteristics of information flow, so that the whole network system can achieve the highest cost performance, and simplify the operation and maintenance steps of the network as much as possible, so that it can be easily managed and maintained.

(3) $$\begin{array}{c} f\left(x^{s}\right)=w^{T}\theta x^{s}-u^{T}x_{1}^{s}. \end{array}$$

**Figure 2:**
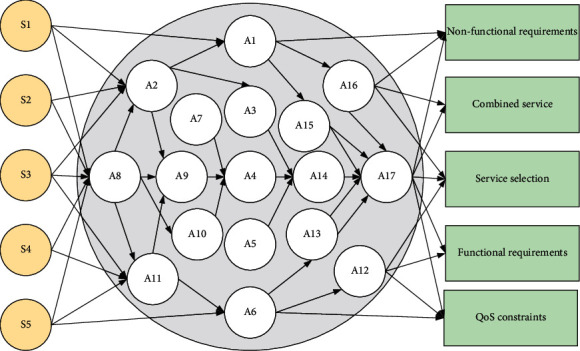
Abstract service framework.

Database conceptual structure design refers to the process of creating an abstract conceptual data model by describing the summarized user requirements. The data model is represented in abstract form. A modeling approach based on the entity-relational model (E.R. model) describes the conceptual design of the database. First, we must understand the actual entities involved in each party, the attributes it contains, the entity relationships, and the interconnection and reference to the way the information is implemented to obtain a partial map of each participant, as well as returning to the multiple partial views obtained. The collection is a global view of the conceptual data model that the user wants to describe and correspond to, as shown in Figure 2.

Smart tourism forms intelligent tourism information through the proficiency in tourism data. This intelligent and symmetrical information is oriented towards not only the tourist, but also the providers of the various supply elements in the tourism industry. The various suppliers in the tourism industry use tourism information to meet consumer demand promptly, interact with consumers through a network platform, and dynamically adjust the business models of individual enterprises to accommodate changes in demand. The orderly flow of tourism information throughout the entire industrial system forms the scattered supply elements into an organic whole, ultimately forming a closely coordinated intelligent tourism ecosystem to meet the diverse needs of tourists [14]. The intelligent tourism ecosystem can actively mobilize elements of value to fully integrate resources and fully understand the real-time and potential tourism needs of tourists to enable the whole system to collaborate effectively; the ecology through the evolution of one species to influence other species and promote the evolution of other species is the whole system of synergistic evolution.

(4) $$\begin{array}{c} \mu_{s}=\frac{1}{n_{1}}\sum_{i=1}^{n}\theta x_{i}^{s2},\\ \max\frac{1}{2}{\left\|\mu_{s}^{\pi }-\mu_{t}^{2}\right\|}_{2}^{2}=\frac{1}{2}\left|\frac{1}{n_{1}}\sum_{i=1}^{n}\theta x_{i}^{s2}-\frac{1}{n_{1}}\sum_{j=1}^{n2}\theta x_{j}^{s2}\right|. \end{array}$$

The primary stage of smart tourism is still based on the latest information and network technology to enhance the information level of tourism supply entities. The limitations of the vast number of small and medium tourism enterprises themselves in terms of information technology investment and the lack of talents make it difficult to keep pace with the development of smart tourism and are easily marginalized. It aims to establish a systematic and scientific intelligent tourism service management theory and method system based on the network platform as the carrier and technical support, to improve the collaborative service ability of the entire tourism industry and further enhance the comprehensive advantages of the tourism industry. Specifically, free information technology solutions can be provided first, with subsequent revenue distribution with the network platform determined according to the business performance and scale of the enterprise.

Smart tourism information interaction services also require some professional supporting services, such as smart navigation service modules, which have special organizations with relevant information, and the network platform and tourism companies are not good at carrying out such professional services but need to purchase some professional database or supporting services to achieve the information supply service function.

(5) $$\begin{array}{c} \text{s}.\text{t}.\sum_{k\in N_{i}^{x}}w_{ik}^{s}=2. \end{array}$$

From the perspective of the network platform tourism information query function, it is necessary to form modular service information for the query according to consumers' possible wisdom tourism whole process information service needs, such as wisdom guide, wisdom navigation, a wisdom shopping guide, and other kinds of specific functions; at the same time, according to the network platform tourism information advantages and the general tourists' common and individual needs, through the module combination to form different tourism program information for the query, service matching is the interaction process between tourists and the query interface of the network platform, according to the query demand for complete information docking belongs to complete matching, for the inclusion of matching or partial matching the formation of the priority order of the network platform recommendation suggestions. For matching failure, it should also form certain feedback as well as information records.

Mainly for the tourism consumption process of the scene and time and space changes in the timely information wisdom service, to wisdom tour guide for example, the network platform has formed a typical wisdom tour guide program; if combined with the tourists intelligent positioning, it can be in the scenic spot of different attractions online wisdom tour guide service. Firstly, the key to the network platform programmed services lies in the common needs of the possible information interaction services in the process of consumer tourism for excavation, and the corresponding information service scheme for these needs for optimized design. Secondly, the system development of hardware and software is related to various intelligent terminals and their networked links [15]. Finally, there are the network platform programmed services to achieve the whole process of peer-to-peer docking of tourist demand and tourism supply information.

As a result of the advanced extension of tourism informatization, intelligent tourism is formed and reflects the following three characteristics, namely, humanization, personalization, and intelligence [16]. It is important to recognize that the goal of tourism informatization is clear, that is, the development of informatization, analyzed in terms of tourism enterprises or destinations, although smart tourism is different, analyzed from the perspective of the tourist, and more concerned with the experience gained by the visitor, allowing the humanizing features to be manifested. The biggest advantage of smart tourism is that it enables the different needs of each visitor to be met, with different products being designed for different travelers. Smart tourism can bring out the subjective initiative of the tourist and put into practice a truly intelligent experience throughout, and these goals are achieved based on tourism information technology.

The combination of a wide range of high technologies offers the possibility of intelligent tourism, enabling huge amounts of information to be interacted with, facilitating the tourist, and enabling them to make better and more rational plans to meet a variety of travel needs and to have a better experience.

### 3.2. Analysis of Tourism Management Strategies

Tourism destination and scenic area management in the wisdom of tourism government management accounted for greater weight, the wisdom of tourism destination management more into the content of the wisdom of the city management, for the management of scenic areas more clearly reflected in the wisdom of tourism government management platform, so this section focuses on the application of cloud computing in the wisdom of tourism government in the management of scenic areas. The elements of the scenic area include scenic attractions, scenic business entities, tourists, residents, and government departments, such as a few thousand subjects [17]. As the scenic resources are owned by the state, the government has the right to regulate and supervise the management behavior of scenic areas and formulate corresponding policies and regulations. The main content of scenic area management includes scenic area resource management, scenic area environment management, scenic area reception service facilities planning, scenic area visitor guidance and management, scenic area and resident's relationship management, scenic area standardization and quality management, and safety management. Scenic resource management includes tourism resource management, land resource management, human resource management, and information resource management. The information collection accessed by the cloud computing wisdom tourism government management platform is mainly in information resource management, through the collection and processing of scenic database data to analyze the operation of all aspects of the scenic area [18].

**Figure 3:**
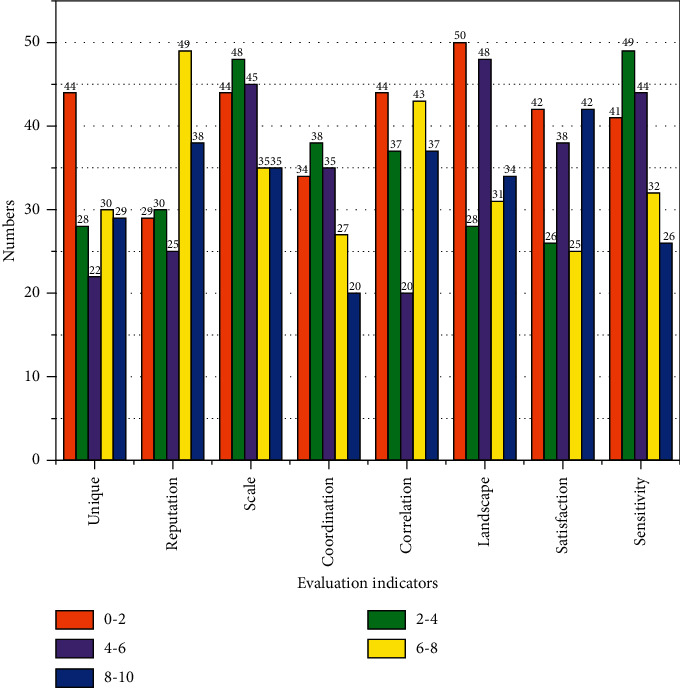
Scoring criteria for comprehensive evaluation of ecotourism development.

Scenic environmental management is mainly from the tourism environment capacity and tourism environment evaluation of two aspects of management, through the scenic monitoring equipment, such as image monitoring equipment, water quality monitoring equipment, and other equipment to monitor the changes in the scenic environment, and through the scenic information system docking to the corresponding cloud computing data center library of the intelligent tourism management platform, so that management departments can perform real-time monitoring and early warning, such as mudslides before the outbreak of water quality. The rapid changes in water quality before the outbreak of mudslides are, for example, sent to tourists and the corresponding departments through the cloud computing platform in time to evacuate tourists. Scenic service facilities and reception management include planning and management of food, accommodation, travel, tourism, shopping, and entertainment facilities in scenic areas; real-time information on hotel room reservations in scenic areas can be synchronized with the intelligent tourism government management platform and the intelligent tourism public service platform, monitoring information on the health and safety of food and service facilities in scenic areas combined with the visitor complaint platform, and statistics on the usage rate and several people in recreational facilities in scenic areas are beneficial to the management and marketing of scenic areas. The usage rate and several people in the recreational facilities in the scenic area are conducive to the management and marketing decisions, and the usage of the facilities in the scenic area is conducive to the planning and optimization of the number of people in the scenic area, as shown in Figure 3.

The target end-customers are marketed through the platform to enter the scenic area, which contains both public and commercial services, which fits well with the conclusion of the cloud-based intelligent tourism public service platform and government management platform discussed in the thesis. According to the comprehensive evaluation model of ecotourism development of the Grassland Skyway constructed above, and taking into account the scores and weights of various indicators, the comprehensive evaluation score of ecotourism development of the Grassland Skyway is calculated to be 5.409 < 6.0, and according to the evaluation standard of ecotourism development of the Grassland Skyway, the ecotourism development of the Grassland Skyway is currently in the pan-ecotourism stage, which is the initial stage of ecotourism development [19]. This stage is the initial stage of ecotourism development, in which the ecological concept is applied to mass tourism, and the degree of protection of ecotourism resources in the process of tourism activities is poor, indicating that the ecotourism development of the Grassland Skyway is far from the standard.

**Figure 4:**
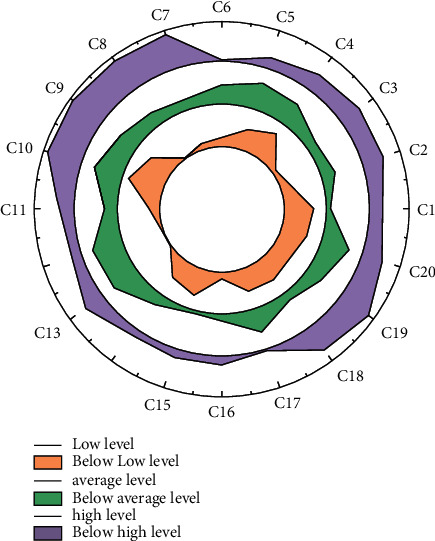
Normalization of survey data for evaluation indicators.

From the evaluation results, the Grassland Skyway's tourism landscape environment, natural ecological environment, and product competitiveness score high, indicating that it is rich in ecotourism resources, has a good ecological environment, and has a good foundation for the development of ecotourism, providing the necessary conditions for the reasonable development of ecotourism activities. In this way, the pain points and needs of tourists in scenic spots can be solved, and the whole journey of tourists in scenic spots can be made more intelligent, humanized, featured, and refined. In contrast, its human tourism environment, development behavior, residents' behavior, and eco-economic benefits score lower. Analysis of the reasons for this is mainly due to the late start but rapid development of tourism on the Grassland Skyway, the weak penetration of the eco-tourism concept in terms of tourism environment construction and protection, the guidance of tourism participants' behavior, tourism products, and benefits, especially the low degree of implementation of the eco-tourism concept in the development process, in addition to the government's attention. The degree is not enough, the capital investment is seriously insufficient, there is a lack of reasonable business management model, the infrastructure of catering, accommodation, and transportation needs to be further improved, the supply of tourism service specialists is insufficient, the service quality is low, the residents participate in the development of ecotourism in an incorrect way and with few opportunities, and good ecological and economic benefits have not been achieved, as shown in Figure 4.

Through the above analysis and calculation process, the shortcomings and advantages of wisdom tourism in each indicator can be intuitively seen as follows: in terms of wisdom tourism basic service facilities, the proportion of evaluation of the high and very high level of IoT construction is significantly lower than that of other indicators, indicating that more efforts are needed in IoT construction; in terms of wisdom tourism management, the proportion of evaluation of the high and very high level of wisdom tourism talents is significantly lower than that of other indicators; and in terms of smart tourism marketing, the evaluation ratio of low level of personalized marketing and new media marketing is high, indicating that there are shortcomings in these two aspects; in terms of smart tourism main enterprises, the evaluation ratio of low level of retail wisdom is significantly higher than that of other indicators, indicating that there is room for improvement in the wisdom of tourism shopping [20]. At the same time, the proportion of smart tourism scenic spots with an overall rating above high is significantly higher than that of others, indicating that more good achievements have been made in the construction of smart tourism scenic spots; in terms of the smart tourism participant population, the overall rating is evenly above high, indicating that the quality of the population, networking, and smart tourism participation is good; in terms of smart tourism quality perception, all parts of the indicators show high performance, indicating that tourists' perception of the overall evaluation of smart tourism tends to be good, and the expectation is high.

The data analysis system, which not only supports the statistical analysis function of the current scenic business data, but also plays a future role in docking other business systems within the scenic area, forming a tourism big data circle to exchange with each other, effectively drives the overall information development of the scenic area, thus rapidly improving the management and service level of the scenic area, which can compete for additional merits and be applied for a higher level of scenic area assessment.

Travel agency management system includes travel agency establishment, travel agency change registration matters or termination of operation, travel agency setting up branches for the record, travel agency information filling, and outbound tour management system. The application of cloud computing in the management of travel agencies includes the uploading and updating of travel agency qualification materials and the filing of travel agency information under the trend of paperless e-government, the social supervision mechanism of the services provided by travel agencies through the public service platform of intelligent tourism, and the notification of information on travel agencies' violations of law and regulations. The application of cloud computing in the management of travel agencies also includes the collection of statistics on the data received by travel agencies and the departure of tours, and the statistics on the number of inbound and outbound tours can be used for holiday travel trends, the effective allocation of corresponding resources, the use of the number of groups booked to estimate the number of people received by tourist attractions, and the effective allocation of corresponding tourist destinations and scenic spots.

## 4. Analysis of Results

### 4.1. Smart Tourism Platform Performance Results

The process of decoding the basic visitor information, data collection, and RFID reader transmission includes the RFID number, name, visitor's ID number, and current location light. The data information is then filtered, encapsulated, and sent to the management center. After receiving the data from the RFID intermediate device, the management center analyzes the data and visualizes it. The ice and snow tourism platform based on RFID anticollision algorithms can be used for the management of scenic areas across the region and enables the intelligent management of different elements such as tourists, scenic areas, tour operators, and guides.

**Figure 5:**
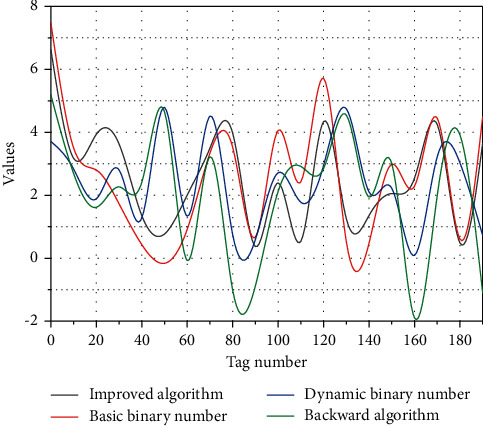
An algorithm for calculating accuracy with the number of actuarial calculations.

In terms of the reliability of the smart tourism platform in the rating table and the accuracy of the RFID identification, the test values of these two indicators are within the design range and fully meet the expectations in scenic spot ticketing system management, environmental monitoring, scenic spot traffic management, scenic spot entertainment management, scenic spot office management, and visitor management. In particular, the three areas of ticketing system management, scenic traffic management, and attraction visitor management are well above these two indices. This indicates that these three main management systems ensure the reliability of the above indices. RFID anticollision algorithms are then tested, and to describe the advantages of RFID anticollision algorithms, three other algorithms, the basic binomial tree algorithm, the dynamic binomial tree algorithm, and the inverse algorithm, were used as control groups, and the test results are shown in Figure 5. All four algorithms are extremely stable, and the accuracy of the results of all four algorithms increases with the increase in computation time, but the RFID accuracy and RFID anticollision algorithms perform best in time.

According to the conditions of applicability of the wisdom tourism service model, the characteristics of the dominant advantages of ice and snow tourism services, and the stage of development of the tourism industry, we choose to build an ice and snow tourism platform as the consumer tourism entrance, realize the construction of an ice and snow wisdom tourism service network based on a network platform, and design a collaborative service model of ice and snow wisdom tourism elements to cultivate and consolidate the dominant advantages of ice and snow wisdom tourism. The ice and snow tourism platform as a consumer portal can provide a series of services based on ice and snow resources such as smart travel, smart maps, smart weather, smart food, and smart hotels. The platform itself can integrate certain tourism elements and can provide standard services including six types of elements including “food, accommodation, travel, shopping, and entertainment” as well as accurate recommendation services and customized services according to consumers' tourism needs.

The itinerary planning module is divided into three submodules: itinerary planning, classic tour route, and attraction guidance. The itinerary planning module plans and recommends itineraries for tourists based on their current location, tour time, and tour budget in the scenic spot. After preview view, itinerary guidance, classic tour line submodule is the scenic area according to the tourists' tour strategy and experience, organized out of the classic route recommendations, and tourists can preview the line and line guidance; attraction guidance function is mainly for the tourists familiar with the scenic area, and in the default state of the system department for the path guidance service, tourists use the free-flowing guide mode, and the system will not provide each intersection guidance voice. The system will not provide voice prompts for guidance at intersections but will only automatically explain the pathway according to the visitor's real-time location.

**Figure 6:**
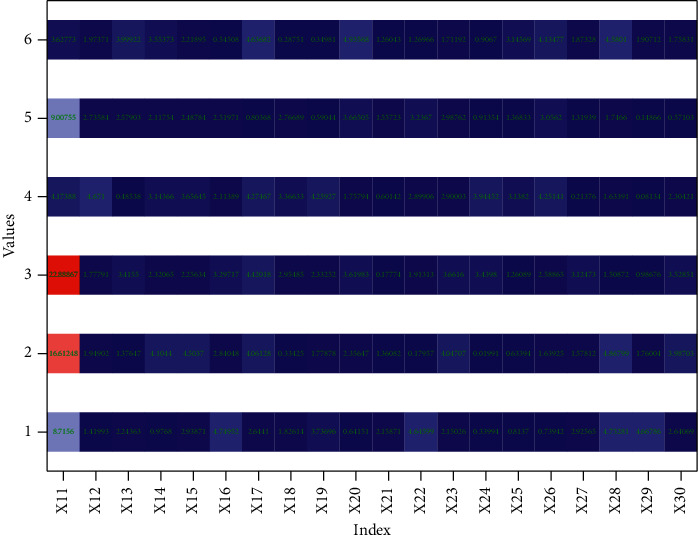
Entropy values and coefficients of variation for each indicator.

System testing is a standard process in the system development process. System testers compare the actual results of the system operation with the expected results through manual or instrumental methods to assess the quality of the system, discover possible problems in the various processes of system requirements, design, or implementation, and fix them at the first time to ensure the stability of the system after it goes online. A single IoT service cannot meet user needs well, so service composition technology is particularly important. Service composition is a process of aggregating multiple services with a simple structure and a single function into a composite service process with a complex structure and powerful functions through a certain combination mode. At the same time, to verify that the system meets the performance requirements and that the system can run stably, it is necessary to carry out diverse tests on the performance of the system by setting the relevant conditions according to the system performance requirements. In this paper, we will combine the nonfunctional requirements of the system, mainly from the implementation of system functions, system performance requirements, and system compatibility requirements to test and verify this system, as shown in Figure 6.

The orderliness between the three constituent dimensions of tourists, tourism elements, and supply and demand matchmakers shows that the orderliness of tourist participation and cooperation is the greatest, indicating that tourist participation in Harbin Ice and Snow Smart Tourism is high, but the orderliness of the supply and demand matchmakers' dimension is low.

It is important to note that, due to the influence of network technology, the degree of elemental cooperation, and the degree of ice and snow tourism resource aggregation, the value cocreation service model of Harbin Ice and Snow is not yet in operation, while the information interaction and elemental cooperation service model is the current main service model of Harbin Ice and Snow Smart Tourism. In the future, along with the development of various information technologies such as artificial intelligence, the Internet of Things, and virtual reality, the role of network platforms in resource aggregation, elemental integration, and service innovation will be further highlighted, and the value of various tourism participants will be cocreated.

### 4.2. Tourism Management Strategy Results

This is a good boost to the entry of the Grassland Skyway Management Office, so the higher government should seize this opportunity and give the Grassland Skyway Management Office more management authority, so that they can establish a good development environment (including soft and hard environment) through several years of management and trial. For the sustainable development of grassland Tianqi ecotourism, a perfect supervision system must be established to regulate the duties of the grassland Tianqi Management Office, so that the application of the rights of the grassland Tianqi Management Office is systematically guaranteed and managed. Making the Grassland Skyway Management Office a civilized unit with transparent and standardized management that is iconic in Zhangjiakou and even in the province is also a way of protecting all the staff of the Grassland Skyway Management Office. Through regulation, they will be able to better enforce the law and deal with related matters according to the law.

**Figure 7:**
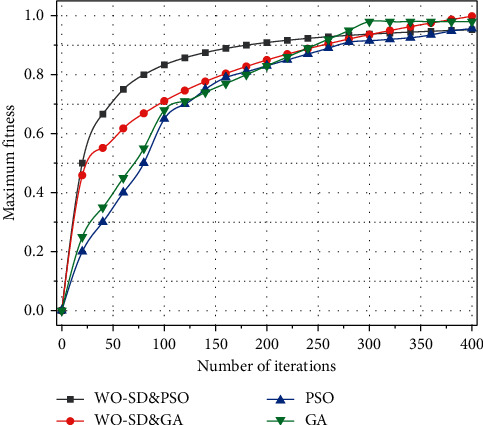
Maximum adaptation value for different iterations.

Through the development of relevant preferential policies to increase foreign investment, to attract social capital into the development of the grassland sky road ecological tourism resources, and to enhance the grassland sky road tourism brand taste, there is the implementation of the side investment, side development of the input-output model, and the formation of tourism to raise tourism virtuous cycle of development mode, but also to actively provide a good development environment for the development of enterprises. Relevant tourism enterprises should develop themselves by changing their thinking, gradually developing towards scale, specialization, and grouping and improving the business capacity of enterprises, as shown in Figure 7.

Combining all the above experiments, the results show that, in large-scale service combination instantiation, compared with the traditional service combination method, based on different sets of weight factors, different QoS models, and different heuristic algorithms, the service combination method based on service domination proposed in this chapter can significantly reduce the service combination scale of the workflow, thus improving the service combination efficiency and service combination quality.

In the aspect of intelligent tourism infrastructure service facilities, the proportion of evaluation of high and very high level of IOT construction is significantly lower than that of other indicators, indicating that more efforts are needed in IOT construction; in the aspect of intelligent tourism management, the proportion of evaluation of high and very high level of intelligent tourism talents is significantly lower than that of other indicators, and the proportion of evaluation of low level is significantly higher than that of other indicators, indicating that talent construction is a more strongly reflected prominent. In terms of smart tourism marketing, the proportion of evaluation of low level of personalized marketing and new media marketing is high, indicating that Luoyang City has shortcomings in these two aspects; in terms of smart tourism main enterprises, the proportion of evaluation of low level of retail wisdom is significantly higher than that of other indicators, indicating that there is room for improvement in the wisdom of tourism shopping, while the proportion of overall evaluation of smart tourism scenic spots above high is significantly higher than that of other methods, indicating that Luoyang City has a higher proportion of evaluation of smart tourism scenic spots in general than other. It shows that Luoyang City has achieved relatively good results in the construction of smart tourism scenic spots; in terms of smart tourism participating people, the overall evaluation is evenly above high, indicating that the quality of people, networking, and smart tourism participation are good; in terms of smart tourism quality perception, all partial indicators show high results, indicating that tourists' overall evaluation of smart tourism in Luoyang City tends to be good, and their expectation is high.

## 5. Conclusion

This paper takes the current situation of the tourism industry as the specific background, studies and analyzes the demand of intelligent tourism system, combines relevant theoretical knowledge, and uses computer technology and software engineering technology foundation and object-oriented development method to complete the demand analysis and system of the intelligent tourism system. Based on the analysis of the existing intelligent tourism system, the design idea of the system is proposed. UML use case diagrams are used to complete the analysis of the functional requirements of the system, and use case specifications are given. In the system design, the system functional architecture is analyzed, the functional structure diagram of the system is given, and the class diagram and sequence diagram design of each functional module of the system are given, while the entities of the system and their relationships are analyzed, the entity-relationship diagram is drawn, and the design of the database table structure is given; and finally, the intelligent tourism system is implemented. The design architecture and ideas of the collaborative service model of wisdom tourism elements are proposed, and three service modes are designed: efficient collaborative service for common realistic needs, precise collaborative service for individual realistic needs, and intelligent collaborative service for potential needs; the network platform-based collaborative service mode of wisdom tourism elements, such as demand identification and mining, resource gathering and optimization, supply and demand matching and tracking, and service feedback and innovation, is constructed. It also proposes a management strategy for collaborative services. In the design process of the whole intelligent travel system, not only the theoretical knowledge is enriched, but also the ability to use tools such as software modeling is improved.

## Data Availability

The data used to support the findings of this study are available from the corresponding author upon request.

## References

[1] Gretzel U., Koo C. (2021). Smart tourism cities: a duality of place where technology supports the convergence of touristic and residential experiences. *Asia Pacific Journal of Tourism Research*.

[2] Corrêa S. C. H., Gosling M. S. (2021). Travelers’ perception of smart tourism experiences in smart tourism destinations. *Tourism Planning & Development*.

[3] Fusté-Forné F., Jamal T. (2021). Co-creating new directions for service robots in hospitality and tourism. *Tourism and Hospitality*.

[4] Alam T. (2021). Cloud-based IoT applications and their roles in smart cities. *Smart Cities*.

[5] Bastidas-Manzano A.-B., Sánchez-Fernández J., Casado-Aranda L.-A. (2021). The past, present, and future of smart tourism destinations: a bibliometric analysis. *Journal of Hospitality & Tourism Research*.

[6] Wang N. (2022). Application of DASH client optimization and artificial intelligence in the management and operation of big data tourism hotels. *Alexandria Engineering Journal*.

[7] Stankov U., Gretzel U. (2021). Digital well-being in the tourism domain: mapping new roles and responsibilities. *Information Technology & Tourism*.

[8] Xiang Z., Fesenmaier D. R., Werthner H. (2021). Knowledge creation in information technology and tourism: a critical reflection and an outlook for the future. *Journal of Travel Research*.

[9] Yang Z. (2021). Analysis of customer-oriented information for tourism enterprises under the social media environment. *Entrepreneurship Research Journal*.

[10] Li C.-Y., Fang Y.-H., Sukoco B. M. (2021). Value proposition as a catalyst for innovative service experience: the case of smart-tourism destinations. *Service Business*.

[11] Jamal T., Higham J. (2021). Justice and ethics: towards a new platform for tourism and sustainability. *Journal of Sustainable Tourism*.

[12] van Nuenen T., Scarles C. (2021). Advancements in technology and digital media in tourism. *Tourist Studies*.

[13] Bhaiswar R., Meenakshi N., Chawla D. (2021). Evolution of electronic Word of Mouth: a systematic literature review using bibliometric analysis of 20 years (2000–2020). *FIIB Business Review*.

[14] De la Mora Velasco E., Huang A., Haney A. (2021). An employee sharing model for the tourism and Hospitality industry. *Tourism and Hospitality*.

[15] Gössling S. (2021). Tourism, technology and ICT: a critical review of affordances and concessions. *Journal of Sustainable Tourism*.

[16] Sotiriadis M. (2021). Tourism destination marketing: academic knowledge. *Encyclopedia*.

[17] Mehraliyev F., Choi Y., King B. (2021). Theoretical foundations of social media power in Hospitality and tourism: a Hierarchical model. *Cornell Hospitality Quarterly*.

[18] Fararni K. A., Nafis F., Aghoutane B., Yahyaouy A., Riffi J., Sabri A. (2021). Hybrid recommender system for tourism based on big data and AI: a conceptual framework. *Big Data Mining and Analytics*.

[19] Gelter J., Lexhagen M., Fuchs M. (2021). A meta-narrative analysis of smart tourism destinations: implications for tourism destination management. *Current Issues in Tourism*.

[20] Toubes D. R., Araújo Vila N., Fraiz Brea J. A. (2021). Changes in consumption patterns and tourist promotion after the COVID-19 pandemic. *Journal of Theoretical and Applied Electronic Commerce Research*.

